# ELIGIBILITY FOR LOW-DOSE COMPUTED TOMOGRAPHY LUNG CANCER SCREENING IN OLDER ASIAN AMERICAN SMOKERS

**DOI:** 10.1093/geroni/igz038.679

**Published:** 2019-11-08

**Authors:** Chien-Ching Li, Kelsey Choi, Alicia Matthews, Raj Shah

**Affiliations:** 1 Rush University, Chicago, Illinois, United States; 2 University of Illinois at chicago, Chicago, Illinois, United States

## Abstract

Lung cancer is the leading cause of cancer-related deaths in Asian Americans. Low-dose computed tomography lung cancer (LDCT) screening is an effective way to decrease lung cancer mortality. This study aimed to examine the difference in LDCT screening eligibility among Asian American subgroups. The National Health Interview Survey data (2006-2016) was analyzed. The U.S. Preventive Services Task Force guideline was used to determine the LDCT eligibility. A higher and statistically significant proportion of current Filipino smokers (35.4%) met LDCT screening eligibility criteria compared to Chinese (26.5%) and other Asian smokers (22.7%) (p=0.02). Hierarchical logistic regression results further showed that Filipino were more likely to meet LDCT screening criteria than other Asian while adjusting demographics (OR=1.87; p=0.01). The differences in LDCT screening eligibility no longer existed after additionally adjusting socioeconomic factors as well as perceived health status. Future targeted outreach and intervention research is needed for Filipinos with lower socioeconomic status.

